# Unraveling the Complex Cellular Repair Mechanisms Following Myocardial Infarction

**DOI:** 10.3390/ijms26136002

**Published:** 2025-06-23

**Authors:** Ruiling Chen, Yalin Fu, Ling Hu, Yuqing Chen, Pengyun Li

**Affiliations:** 1 The Key Laboratory of Medical Electrophysiology of Ministry of Education, Medical Electrophysiological Key Laboratory of Sichuan Province, Collaborative Innovation Center for Prevention and Treatment of Cardiovascular Disease of Sichuan Province, Institute of Cardiovascular Research, Southwest Medical University, Luzhou 646000, China; 20210518330105@stu.swmu.edu.cn; 2 Clinical Medical College, Southwest Medical University, Luzhou 646000, China; 20210619330320@stu.swmu.edu.cn (Y.F.); 20210619330423@stu.swmu.edu.cn (L.H.); 3 School of Public Health, Southwest Medical University, Luzhou 646000, China; 20220619330127@stu.swmu.edu.cn

**Keywords:** myocardial infarction, cellular repair, mechanism, non-cardiac stem cells, cardiac explants

## Abstract

Growing evidence underscores the pivotal roles of both in situ-resident and -non-resident cardiac cells in the repair mechanisms following myocardial infarction (MI). MI continues to be a predominant cause of death and disability, posing a significant threat to global health and well-being. Despite advances in medical care, current therapies remain insufficient in preventing ventricular remodeling and heart failure post-MI. We seek to clarify the underlying regenerative mechanisms by which distinct cell types contribute to the repair of MI injury and to systematically assess the translational potential and therapeutic efficacy of these cell-based approaches in clinical applications. This review conducts a comprehensive analysis of recent research progress on the roles of non-cardiac stem cells in situ and cardiac cells derived from explants in MI repair. These cells contribute to the repair process through multiple mechanisms, including cell proliferation and differentiation, angiogenesis, paracrine signaling, immune regulation and fibrosis modulation. Our analysis reveals the intricate mechanisms of MI repair and highlights the necessity for developing age-specific therapeutic strategies for certain cell types. This review offers novel insights into cell-based treatment for MI and provides a scientific foundation for future clinical trials of cardiac regenerative medicine.

## 1. Introduction

Cardiovascular disease, particularly MI following coronary artery occlusion, remains the leading cause of death and disability worldwide [1]. While older-onset MI accounts for the majority of cases, early-onset MI has been increasingly prevalent in recent years [2]. For patients with MI across different age groups, neither conventional invasive treatments nor conservative management strategies have significantly reduced the risk of cardiovascular death or non-fatal MI, especially in the elderly population. In adults, short-term efficacy for MI treatment is notable, but the long-term prognosis remains dismal. Meanwhile, neonatal MI is associated with an extremely high short-term mortality rate [3]. Given the limited efficacy of current therapies in preventing ventricular remodeling and heart failure, there has been a growing clinical focus on the potential of cell transplantation for the treatment of post-coronary infarction. Based on a homing mechanism, cell transplantation has the potential to effectively reverse ventricular remodeling and address the root cause of heart disease [4]. Clinical studies have demonstrated that long-term-cultured, high-dose stem cell transplantation can improve left ventricular ejection fraction (LVEF) and restore cardiac function after infarction [5], offering a promising new direction for clinical application.

Cellular involvement in MI cardiac injury and repair mechanisms is extremely complex and influenced by a wide range of factors, among which the percentage of repair cells associated with MI and recovery performance varies across different age groups [6,7]. The underlying mechanisms may be related to cellular senescence, persistent DNA damage and chronic inflammation. By interfering with repair cell types and signaling pathways in neonatal, adult and elderly populations, the downstream secretion of growth factors and cytokines that regulate the cardiac microenvironment is altered, which in turn affects the termination of the inflammatory response, the promotion of cell proliferation and differentiation, neovascularization, fibrosis and scar formation, and other repair processes after infarction [8,9,10,11].

Our objective is to elucidate the potential mechanisms of different cells in the repair of MI injury across various ages, thereby evaluating their potential applicability and efficacy in clinical practice. Additionally, it will explore the current obstacles and prospective methods in cell therapy, with the ultimate goal of propelling the field of cardiac regenerative medicine forward. Through a comprehensive synthesis and analysis of recent research advancements, this review aims to provide novel insights into the therapeutic strategies for primary MI and to provide a scientific basis for future research and clinical trials.

## 2. Myocardial Infarction Pathology and Repair Mechanisms Across Different Ages

### 2.1. Neonatal Myocardial Infarction

Neonatal MI is extremely rare and often presents with nonspecific early symptoms. Its etiology is frequently associated with congenital coronary malformations and Kawasaki disease [12]. Considering the distinctive biological properties of neonatal stem cells, such as enhanced pluripotency, minimal cellular senescence, robust cardiac regenerative potential, natural immunosuppressive properties, and elevated metabolic activity, the repair mechanisms in neonates are markedly different from those in other age groups [13,14]. Most notably, neonatal mammals can exhibit transient but considerable cardiac regeneration potential post-infarction, which can fully restore cardiac function. Cardiomyocytes (CMs) can re-enter the cell cycle after MI and regenerate naturally through the activation of mitotic molecular switches, such as telomerase and GATA4, and so on [15,16,17]. Mesenchymal stem cells (MSCs) and endothelial progenitor cells (EPCs) also exhibit robust regenerative potential [18,19]. Epicardial cells can be regenerated through epithelial–mesenchymal transition (EMT), producing endothelial cells (ECs), smooth muscle cells (SMCs), fibroblasts (Fbs), etc. [20]. Meanwhile, macrophages, lymphocytes and other inflammatory cells are recruited. Given that the neonatal immune system remains immature, T-lymphocytes have a propensity to acquire a regulatory phenotype, with the reduced ability of secreting pro-inflammatory mediators and fibrotic responses, thus playing a role in alleviating inflammation and promoting vascular regeneration alongside B-lymphocytes [21,22].

### 2.2. Elderly Myocardial Infarction

In contrast, the elderly population, which has a high prevalence of MI, exhibits a complex and long-lasting post-infarction inflammatory response. The host is in a state of “inflammatory senescence”, characterized by reduced activity of stem cells and a pronounced fibrotic response [19,23]. Owing to telomere shortening, oxidative stress, and other pathological changes, cellular senescence (in fibroblasts, ECs, etc.) is evident, with significant myocyte necrosis and apoptosis. Cardiomyocytes possess minimal regenerative capacity, and ventricular degenerative changes are frequently observed [8,24]. Repair cell proliferation and transformation are inhibited, resulting in insufficient scar formation, which compromises the integrity of the cardiac structure and functional restoration [13]. Cellular senescence is crucial for significantly prolonging calcium decay, which consequently leads to a marked decrease in post-infarction cardiac contractile capacity [25].

### 2.3. Adult Myocardial Infarction

The physiological characteristics of post-infarction repair in adulthood are intermediate compared to those of the neonatal and elderly populations. The mechanisms of cellular repair following MI include bi-directional inflammation-mediated processes, cell-mediated repair, vascular regeneration, and molecular regulation of the cell cycle [11]. At this stage, cardiomyocytes primarily serve as the primary functional cells responsible for essential contraction and pumping functions, which are mainly involved in cardiac repair via non-cardiac in situ-resident stem cells and other immune cells and explant-derived cardiac cell populations [10,26].

**Figure 1:**
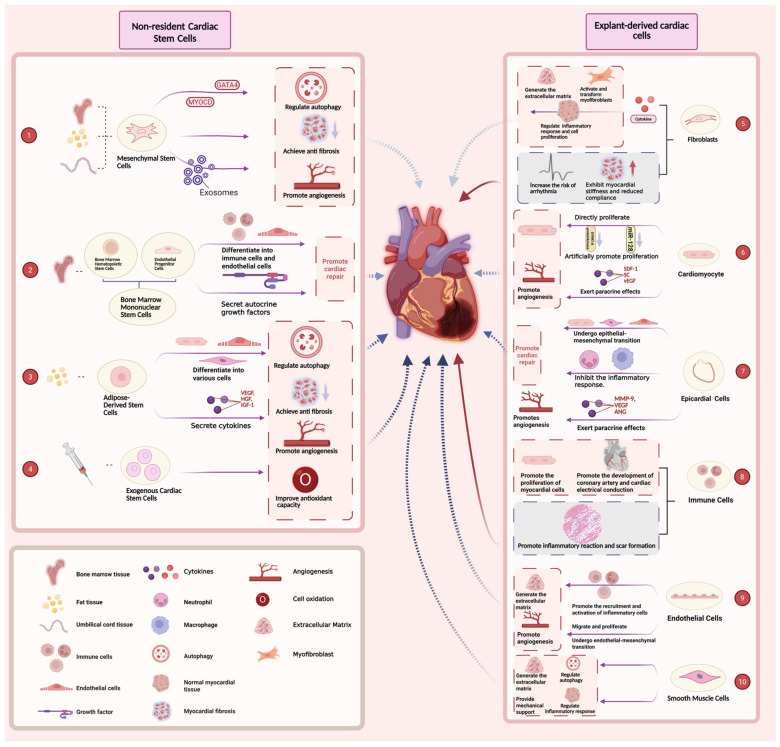
Cellular mechanisms in myocardial infarction (MI) repair. This figure illustrates the roles of various cells in MI repair. Non-cardiac resident stem cells in situ include mesenchymal stem cells (MSCs) from diverse sources: MSCs secrete exosomes to regulate autophagy, achieve anti-fibrosis, and promote angiogenesis via GATA4 and myocardin (MYOCD) (①); bone marrow mononuclear stem cells (BM-MNCs) can differentiate into bone marrow hematopoietic stem cells (BM-HSCs) and endothelial progenitor cells (EPCs), which further differentiate into immune cells and endothelial cells (ECs), which secrete autocrine cytokines to promote cardiac repair (②); adipose-derived stem cells (ADSCs) (from fat tissue) secrete cytokines to regulate autophagy, achieve anti-fibrosis, and promote angiogenesis (③); and exogenous cardiac stem cells (from umbilical cord tissue) also contribute to cardiac repair through similar mechanisms (④). Fibroblasts generate extracellular matrix (ECM), activate myofibroblasts to regulate inflammation and cell proliferation, and increase myocardial stiffness and the risk of arrhythmia (⑤). Cardiomyocytes undergo direct proliferation and secrete paracrine factors like vascular endothelial growth factor (VEGF) to promote angiogenesis and repair (⑥). Epicardial cells promote the epithelial–mesenchymal transition (EMT) and inhibit inflammation while facilitating angiogenesis (⑦). Immune cells promote myocardial cell proliferation and the development of coronary arteries and cardiac electrical conduction (⑧). ECs generate ECM, recruit inflammatory cells, and promote migration, proliferation, and lumen formation to drive angiogenesis and the endothelial–mesenchymal transition (⑨). Smooth muscle cells (SMCs) regulate autophagy, provide mechanical support, and modulate inflammatory responses (⑩). Figure created in BioRender (https://BioRender.com/a7c2og6, accessed on 20 June 2025).

In this paper, we present a comprehensive analysis of the distinctive characteristics and repair mechanisms of different cell types based on recent studies (Figure 1).

## 3. Various Cells in the Heart Participate in the Process of Post-Infarction Injury Repair

MI remains a significant global health challenge, with limited therapeutic options for preventing post-infarction complications such as ventricular remodeling and heart failure [5]. Recent studies have highlighted the potential of cell-based therapies in promoting cardiac repair and regeneration [11]. The heart is a complex organ with diverse cell types, each contributing uniquely to the repair process following MI. Understanding the intricate roles and interactions of these cells is crucial for developing effective regenerative strategies. This section aims to provide a comprehensive overview of the contributions of various cell types, including non-cardiac in situ-resident stem cells and explant-derived cardiac cells, to the repair mechanisms following MI. We will explore how these cells participate in processes such as cell proliferation, differentiation, angiogenesis, paracrine signaling, immune regulation, and fibrosis modulation, which are essential for effective cardiac repair [8,10].

### 3.1. Non-Resident Cardiac Stem Cells

At the beginning of the 21st century, the theory of cardiac c-kit-positive stem cell repair had led to a boom in myocardial regeneration research. However, subsequent investigations confirmed systematic data tampering (e.g., falsifying evidence of cell differentiation) in 31 key papers published by Harvard’s Anversa team between 2001 and 2012, and its central conclusion—that c-kit stem cells can effectively regenerate cardiac muscle—was completely discredited [27]. The incident led to the termination of clinical trials worldwide and prompted a shift in research focus to new directions such as exosomes and cardiac fibroblast reprogramming, as well as the strengthening of mechanisms for reviewing the reproducibility of scientific data.

Despite the significant setback caused by the fraudulent “cardiac stem cell research” incident, the concept of “cardiac stem cells” should not be narrowly confined to in situ-resident stem cells such as “myocardial progenitor cells.” The vital role of non-cardiac stem cells in the mechanism of post-infarction injury repair cannot be ignored.

In this study, the term “Non-resident Cardiac Stem Cells” is used to describe stem cells that do not originate from the heart but possess the capacity to migrate to cardiac tissues and contribute to their repair and regeneration. These cells, typically derived from sources such as bone marrow, adipose tissue, or umbilical cord blood, exhibit multilineage differentiation potential and can give rise to various cell types, including cardiomyocytes, endothelial cells, and fibroblasts [28]. Their therapeutic potential is further enhanced by their ability to secrete paracrine factors that modulate the cardiac microenvironment, thereby promoting endogenous repair mechanisms.

#### 3.1.1. Mesenchymal Stem Cells

MSCs are derived from various tissues, including bone marrow, adipose tissue, umbilical cord, etc. Due to their accessibility, multidirectional differentiation potential, and low immunogenicity, MSCs are considered the ideal candidates for cardiovascular cell transplantation therapy [29,30,31,32]. By regulating cell autophagy and senescence, MSCs can achieve post-infarction mechanisms such as anti-fibrotic effects, inhibition of cardiac remodeling, promotion of neovascularization, and enhancement of antioxidant capacity [29,33,34]. The repair mechanisms of young and aging MSCs differ, primarily attributable to the distinct characteristics of MSCs, signaling pathways, and exosomal regulation mechanisms in each respective period.

Young MSCs exhibit higher mitochondrial membrane potential (ΔΨm) and Nicotinamide Adenine Dinucleotide Hydrate (NADH) levels, lower mtDNA content but higher mitochondrial mass, and stronger proliferation and differentiation capabilities. In contrast, aged MSCs exhibit lower ΔΨm and NADH levels, higher mtDNA content but lower mitochondrial mass, and reduced proliferation and differentiation abilities [35]. In addition, in animal studies, aged MSCs were found to show significant morphological and functional differences compared to their younger counterparts. Aged MSCs displayed enlarged and irregular cell shapes, contrasting the smaller and uniform spindle-like morphology of young MSCs. Moreover, aged MSCs exhibited reduced proliferative capacity, with cell cycle arrest in the G1 phase, leading to diminished self-renewal frequency [36].

During the repair process after myocardial infarction, young MSCs possess robust proliferation and differentiation capabilities. They attenuate inflammation post-infarction by downregulating matrix metalloproteinase gene expression and enhancing the levels and activities of tissue-derived inhibitory factors [33]. Human umbilical cord MSCs genetically engineered to overexpress miR-214 enhance cardiac repair post-infarction through secretion of exosomes, which promote endothelial cell migration, tube formation, and cardiomyocyte survival by targeting PTEN-mediated AKT activation [37]. Some experiments have also indicated that umbilical cord MSCs, characterized by elevated expression of GATA4 and myocardin (MYOCD), can effectively reduce post-infarction cardiomyocytes apoptosis. These cells also enhance LVEF and left ventricular fractional shortening (LVFS), thereby restoring cardiac function. Furthermore, they play a pivotal role in cardiomyocyte development and injury repair [38].

In contrast, aged MSCs exhibit marked post-infarction changes. These include reduced m6A modification levels, upregulated ALKBH5, and increased miR-155-5p expression. Enhanced cellular senescence is also observed, stemming from increased Cyclin-Dependent Kinase Inhibitor 1C (CDKN1C) expression, mitochondrial dynamics, and elevated levels of senescence-associated β-galactosidase (SA-β-gal) activity, p53, and p21. These changes cause the proliferative and exosomal secretion capacities of MSCs during repair to be diminished, accompanied by pronounced cellular senescence characteristics [39,40]. Additionally, aged MSCs exhibit a lower expression of macrophage migration inhibitory factor (MIF), along with reduced levels of key autophagy-associated proteins (LC3-I/II and Beclin1) and increased p62 expression. The activation of autophagy to counteract cellular senescence is less pronounced, and post-infarction angiogenic capacity is decreased [26]. Moreover, the decreased secretion of microRNA-10α (miR-10α) in senescent MSCs and the increased expression of its target gene, krüpple-like factor 4 (KLF4), could induce apoptosis and reduce the post-inflammatory repair effects by activating the KLF4-BAX/BCL2 pathway [19].

#### 3.1.2. Bone Marrow Mononuclear Cells

Bone marrow mononuclear cells (BM-MNCs) contain bone marrow hematopoietic stem cells (BM-HSCs), EPCs, and MSCs [41]. BM-MNCs from patients with acute myocardial infarction (AMI) have been studied for their potential to improve outcomes in patients with ST-segment elevation myocardial infarction (STEMI), but their overall therapeutic impact remains inconclusive [42,43]. BM-HSCs reside in specialized microenvironments, known as niches, within the bone marrow, where they are maintained by secreted factors from ECs and perivascular cells expressing the Leptin Receptor^+^ (LepR^+^). These niches are essential for maintaining hematopoietic equilibrium and orchestrating immune responses [44]. Inflammatory mediators such as S100 calcium binding protein A9 (S100A9) are released following MI, which can rapidly activate the proliferation and differentiation of BM-HSCs into monocytes and macrophages. This response addresses the demand for immune cells required for tissue repair. They participate in the inflammatory repair process through mechanisms such as repairing myocardial cells and promoting angiogenesis [45].

Neonatal BM-HSCs exhibit greater regenerative capacity than their adult counterparts, which is possibly related to a higher expression of the heat shock factor. Coronary infusion experiments with autologous hematopoietic stem cells (HSCs) under single-ventricle physiological conditions have demonstrated that more juvenile stem cells can promote myocardial repair and angiogenic capacity by secreting elevated levels of growth factors and exosomes, thereby improving cardiac function [46]. As individuals age, the host undergoes a transition into a state of chronic, low-grade inflammation. This condition can significantly impact the metabolic and epigenetic status of HSCs, potentially impairing their regenerative capacity and contributing to age-related pathologies. This enhances their responsiveness to inflammatory stimuli and promotes the generation of inflammatory myeloid cells, exacerbating the development of inflammation after MI [47]. However, increasing the expression of inflammatory markers S100 calcium-binding protein A8/A9 (S100A8/A9) can lead to a reduced number and the impaired function of HSCs, negatively impacting the prognosis of cardiovascular disease in older individuals [48].

EPCs are immature ECs with the capacity to differentiate into mature ECs and secrete protective cytokines and angiogenic factors, such as vascular endothelial growth factor (VEGF) and fibroblast growth factor (FGF). They can participate in vascular repair and neovascularization following MI through paracrine and direct differentiation. Furthermore, they possess immunosuppressive properties that can inhibit T cell proliferation and reduce inflammatory response [49,50,51].

During the neonatal period, fetal sex and maternal glucose metabolic status can influence fetal vascular health and future cardiovascular disease risk by affecting fetal EPC function [52]. Neonatal EPCs typically have lower senescence characteristics, such as lower cyclin-dependent kinase inhibitor 2A (p16INK4a) expression and a longer telomere length, which protects chromosomes from damage [49]. As chronological progression occurs, the number and function of EPCs progressively decline, thereby impairing their role in vascular repair processes [14].

#### 3.1.3. Adipose-Derived Stem Cells

Adipose-derived stem cells (ADSCs) are a category of stem cells with transplantation potential that can restore cardiac function via direct differentiation into cardiomyocytes, vascular SMCs, and ECs [53]. In addition, ADSCs facilitate cardiac regeneration through the secretion of specific cytokines, including VEGF, hepatocyte growth factor (HGF), and insulin-like growth factor-1 (IGF-1). These factors promote vascular neovascularization, reduce apoptosis, and inhibit fibrotic processes, thereby contributing to tissue repair and functional recovery [53,54]. Studies have demonstrated that ADSCs exhibit multipotency, possessing the capacity to differentiate into cardiomyocytes for cardiac repair. Specifically, implantation of CD29-positive brown adipose tissue stem cells (BADSCs) into the infarcted area can replace neoplastic cardiomyocytes, reduce infarct size, and improve left ventricular function [22,55].

ADSCs are a category of stem cells with transplantation potential that can restore cardiac function via direct differentiation into cardiomyocytes, vascular SMCs, and ECs [53]. Animal studies have shown that ADSCs can improve cardiac function after myocardial infarction. For example, Kashiyama et al. [53] demonstrated that an adipose-derived stem cell sheet under an elastic patch significantly improved cardiac function in rats after myocardial infarction. These studies highlight the therapeutic potential of ADSCs in promoting cardiac repair and regeneration.

ADSCs also facilitate cardiac regeneration through the secretion of specific cytokines, including VEGF, HGF, and insulin-like growth factor-1 (IGF-1). These factors promote vascular neovascularization, reduce apoptosis, and inhibit fibrotic processes, thereby contributing to tissue repair and functional recovery [54]. Clinical trials have demonstrated that ADSCs exhibit multipotency, possessing the capacity to differentiate into cardiomyocytes for cardiac repair [22]. Specifically, animal experiments have shown that implantation of CD29-positive BADSCs into the infarcted area can replace neoplastic cardiomyocytes, reduce infarct size, and improve left ventricular function [55]. Additionally, a meta-analysis by Damianos et al. [56] reviewed the efficacy of adipose-derived stem cell-derived exosomes in preclinical animal models of acute myocardial infarction, showing significant improvements in left ventricular ejection fraction, fractional shortening, infarct size, and fibrosis area, as well as reduced levels of inflammatory cytokines TNF-α and IL-6 and increased levels of anti-inflammatory cytokine IL-10 [56].

#### 3.1.4. Exogenous Cardiac Stem Cells

Exogenous cardiac stem cells can be categorized into ESCs with induced pluripotent stem cells (iPSCs) and adult stem cells. Embryonic-derived stem cells can ameliorate various neonatal diseases by secreting anti-inflammatory factors and promoting vascularization [56]. For instance, embryonic stem cells derived from the bone marrow of neonatal pigs exhibit characteristics typical of pluripotent stem cells (PSCs), which can regulate the biological behavior of cardiomyocytes and fibroblasts, inhibit the fibrotic process, and promote myocardial repair [57]. Early vascular cells derived from embryonic stem cells can self-assemble to form a three-dimensional vascular network. When bioprinted or co-cultured with cardiomyocytes in cardiac microtissues and cardiac patches, they generate an organized microvasculature and show superior self-assembling vascularization capacity compared to individual ECs [58].

The lysophosphatidic acid receptor 4 (LPAR4) is transiently expressed during the differentiation of PSC-derived hearts and is essential for the cardiac progenitor cell stage. Cardiac progenitor cells transiently express SRY-box transcription factor 17 (SOX17) in the embryonic heart, whereas hematopoietic cells (SOX17^−^) migrate from the liver to the bone marrow. By contrast, in adult mouse hearts, after MI, two types of LPAR4^+^ cells were identified by bone marrow transplantation experiments: cardiac-resident LPAR4^+^ cells (SOX17^+^), which possess some cardiac differentiation potential, and bone marrow-derived LPAR4^+^ cells (SOX17^−^), which function only as inflammatory cells and are not involved in cardiac repair [59].

iPSCs are pluripotent stem cells reprogrammed from mature somatic cells, possessing the ability to self-renew and differentiate into various cell types. By differentiating iPSCs into cardiomyocytes, they can be used to repair damaged cardiac tissue. Karbassi et al. utilized CRISPR/Cas9 technology to genetically edit iPSCs, knocking out the genes encoding slow skeletal muscle and cardiac troponin I, thereby generating non-contractile cardiomyocytes. These non-contractile cardiomyocytes, upon transplantation, were able to prevent further deterioration of cardiac function, indicating that paracrine mechanisms may play a significant role in cardiac protection [60]. In addition, iPSCs can be employed for disease modeling and drug screening, providing a platform for investigating the pathogenesis of myocardial infarction and screening therapeutic agents. Through genetic editing techniques, pathogenic gene mutations in iPSCs can be corrected to generate healthy cells for disease treatment [61].

Encapsulating VEGF protein in engineered exosomes can enhance its concentration in damaged myocardial tissue, promoting angiogenesis and cardiomyocyte survival. Sano et al. employed the CRISPR/dCas9 system to activate endogenous genes in cardiac sphere-derived cells (CDCs), augmenting their ability to differentiate into cardiomyocytes. By activating specific cardiomyogenic differentiation factors (such as Gata4, Mef2c, Nkx2-5, Hand2, and Tnnt2), the differentiation efficiency of CDCs was significantly improved, with substantial improvements in cardiac function observed in animal models [61]. Lin Jiang et al. utilized CRISPR technology to activate endogenous genes in fibroblasts, reprogramming them into cardiovascular progenitor cells for myocardial infarction therapy [62].

Engineered exosomes, as a novel therapeutic modality, have also demonstrated potential in myocardial infarction treatment. Exosomes are nanoscale vesicles secreted by cells, containing biomolecules such as proteins, lipids, and nucleic acids. By encapsulating therapeutic molecules in engineered exosomes, drug efficacy can be enhanced and side effects reduced. For instance, encapsulating VEGF protein in engineered exosomes can increase its concentration in damaged myocardial tissue, promoting angiogenesis and cardiomyocyte survival [63].

In addition to the differentiation ability of the above stem cells, there are relevant studies that have demonstrated that cardiac ECs can repair the heart through endothelial-hematopoietic transition [64]. In addition, fibroblasts, BM-MNCs, cardiac fibroblasts and others exhibit a certain degree of differentiation potential. These cells play vital roles in the development and repair of the adult heart [65].

In summary, among the stem cell transplantation therapies, MSCs possess the optimal overall performance. Compared with other cell types, they are characterized by strong immune regulatory capabilities and the secretion of a rich array of factors. Most notably, their therapeutic effects are prominent, effectively suppressing the inflammatory response following myocardial infarction, promoting the polarization of M2-type macrophages, and reducing the infiltration of inflammatory cells. Compared with bone marrow mononuclear cells and adipose-derived stem cells, the likelihood of inducing inflammatory responses is lower [66]. However, the low survival rate of transplanted MSCs in the inflammatory microenvironment, the functional differences between MSCs from different sources, and the resulting potential for unstable therapeutic effects remain significant challenges to be addressed in the future [31].

BM-MNCs are widely used in clinical research, with their low immunogenicity and a certain degree of safety and efficacy in the treatment of acute myocardial infarction [67]. Although BM-MNCs are abundant, their procurement is invasive, and their proliferative capacity is limited. They can still cause immune rejection in allogeneic transplantation, leading to relatively unstable therapeutic effects [68]. ADSCs are easily obtained and have strong differentiation potential, being capable of differentiating into adipocytes, osteoblasts, and chondrocytes [69]. However, their survival rate after transplantation is low, and they may be activated in the ischemic and hypoxic microenvironment to release inflammatory factors, interact with inflammatory cells, and thereby exacerbate the inflammatory response, resulting in inconsistent therapeutic outcomes [70].

### 3.2. Explant-Derived Cardiac Cells

Explant-derived cardiac cells (EDCs) are a population of cells isolated, cultured and expanded from cardiac tissue explants, which have the properties and functions of cardiac tissue. EDCs mainly include cardiomyocytes, cardiac fibroblasts, smooth muscle cells, endothelial cells, etc. [71]. These cells are not only pluripotent, but also secrete a variety of bioactive molecules, such as cytokines and exosomes, which have anti-myocardial, smooth muscle and endothelial properties. These cells are not only pluripotent and capable of differentiating into cardiomyocytes, smooth muscle cells and endothelial cells under specific conditions, but also secrete a variety of bioactive molecules, such as cytokines and exosomes, which have anti-inflammatory, antioxidant, pro-angiogenic and immune-regulating effects [72].

#### 3.2.1. Fibroblasts

After MI, the heart undergoes a complex series of repair processes in which fibroblasts and their activated functional forms, myofibroblasts, serve as pivotal contributors. Fibroblasts represent one of the predominant non-cardiomyocyte cell populations within the heart, and their primary function is to synthesize and secrete extracellular matrix (ECM) to maintain the structural integrity of the heart [73,74]. Following MI, fibroblasts are activated and transformed into myofibroblasts with contractile properties, a critical process that prevents ventricular wall rupture [75].

Prior research has established that fibroblasts, post-MI, play a role not merely in fibrotic scar formation, but also in modulating the electrophysiological properties of the heart via diverse mechanisms. Vasquez et al. found that fibroblasts in the infarcted area exhibit enhanced electrophysiological activity compared with normal fibroblasts, which increases electrical coupling with cardiomyocytes, potentially shortening their action potentials and altering conduction velocities, thereby raising the risk of arrhythmias [73]. Furthermore, fibroblasts exhibit heterogeneity during repair after MI. Fibroblast activation and functional changes are influenced by their developmental origin, whereas the injury microenvironment also plays a significant role [76]. During repair after MI, fibroblasts regulate the inflammation, cell proliferation, and ECM remodeling by secreting various cytokines and chemokines [74]. In recent years, researchers have explored the potential of direct reprogramming fibroblasts into cardiomyocytes to promote heart regeneration. This approach is expected to reduce fibrotic scar formation while restoring the function of a damaged myocardium. However, current reprogramming efficiency remains limited, necessitating further optimization to enhance clinical feasibility [76].

Fibroblast activation during the repair process following MI is also intimately associated with the development of cardiac fibrosis. Talman and Ruskoaho noted that the fibrotic response after MI can be categorized into alternative fibrosis (scar formation) and reactive fibrosis [77]. Alternative fibrosis prevents the rupture of ventricular walls by forming a fibrotic scar. In contrast, reactive fibrosis occurs in non-infarcted areas and results in myocardial stiffness and reduced compliance, ultimately impacting cardiac function. Research has revealed that fibroblast activation and fibrotic responses are governed by multiple signaling pathways, including the TGF-β signaling pathway and the RAS system [74]. In addition, a study by Duan et al. demonstrated that the Wnt1/β-catenin signaling pathway activates epicardial and myocardial fibroblasts following MI to promote cardiac repair, highlighting its key significance in cardiac injury repair and providing a theoretical basis for the development of new therapeutic strategies [78].

Fibroblasts have a multifaceted role in injury repair after MI, maintaining the structural integrity of the heart through fibrotic scar formation and influencing cardiac function by regulating electrophysiological properties and intercellular signaling. Future studies need to further explore the specific mechanisms of fibroblasts in cardiac repair and develop novel fibroblast-targeted therapeutic strategies to improve repair outcomes and cardiac function after MI.

#### 3.2.2. Cardiomyocytes

MI primarily results from ischemic necrosis of the myocardium due to coronary artery obstruction. Its core pathological mechanism involves the massive loss of cardiomyocytes and subsequent fibrotic scar formation. As the main contractile unit of the myocardium, adult cardiomyocytes exhibit extremely limited regenerative capacity after injury, rendering tissue repair and functional recovery after MI a great challenge. However, recent studies have gradually revealed the potential mechanism underlying the role of adult cardiomyocytes in the repair of MI injury, providing a new direction for cardiac regenerative medicine.

In adult mammals, cardiomyocytes undergo cell cycle arrest early after birth, leading to minimal regenerative capacity. However, recent studies have shown that the hearts of neonatal mice and large mammals (e.g., pigs) retain some regenerative capacity for a short period of time after birth, and that their myocardial damage can be partially regenerated by transient proliferation of cardiomyocytes [79].

Cardiomyocytes contribute to the repair process after MI through direct proliferation, but also via their paracrine function. Studies have shown that cardiomyocytes are capable of secreting a variety of cytokines and growth factors, such as stromal cell-derived factor 1 (SDF-1), stem cell factor (SCF), and VEGF, which promote vascular neovascularization, regulate inflammatory responses, and improve the myocardial microenvironment [80].

Electrophysiologic integration of adult cardiomyocytes is essential for restoring normal rhythm and contractile function [81]. Modulation of the cell cycle of adult cardiomyocytes is one of the key strategies to achieve myocardial regeneration. Zhu et al. significantly facilitated cell cycle re-entry of adult cardiomyocytes and improved fibrosis and cardiac function post-MI by knocking down miR-128 [79].

Although the potential of adult cardiomyocytes in MI repair has been partially revealed, their clinical application still faces many challenges, such as cell source, immune rejection and electrophysiological integration [82]. Currently, iPSC technology offers the possibility to solve these problems; induced pluripotent stem cells-derived cardiomyocytes (iPS-CMs) have the advantage of autologous transplantation, preventing immune rejection, and their regenerative ability can be further enhanced by gene editing or pharmacological treatment [81]. Lan et al. significantly improved the proliferation and myocardial repair of transplanted cells by overexpressing CCND2 in iPS-CMs [79]. Meanwhile, inhibiting dual-specificity tyrosine-regulated kinase 1A (DYRK1A) protein kinase significantly activates the cell cycle of cardiomyocytes, thereby improving functional recovery after MI, potentially through mechanisms involving the regulation of histone modifications and transcriptome reprogramming [83].

In the field of biomaterials, researchers have developed a variety of novel materials to promote myocardial repair. Ye et al. developed a hydrogel based on MXene Ti_2_C, which significantly improved functional recovery after MI via its electrical conductivity and biocompatibility [84]. Ren et al. developed an MXene-based conductive hydrogel that significantly improved functional recovery pos-MI by scavenging reactive oxygen species (ROS) and restoring myocardial electrical conduction [85].

The formation of cardiomyocytes plays a crucial role in the repair of MI injury. This process encompasses multiple mechanisms, including cell regeneration, paracrine signaling, electrophysiological integration, and cell cycle regulation. However, the current understanding of these mechanisms is still incomplete. Future research should focus on elucidating the synergistic effects of these mechanisms and develop more effective clinical strategies to achieve efficient tissue repair and functional recovery after MI.

#### 3.2.3. Epicardial Cells

Epicardial cells have been shown to differentiate into multiple cardiovascular cell types, including cardiomyocytes, SMCs, and fibroblasts, through the process of EMT, thereby directly contributing to the regeneration and repair of cardiac tissues through a Bmp2-Smad1/5/8 signaling pathway-dependent manner [86]. In addition, Wang et al. found that the epicardial-specific knockdown of cerebral cavernous malformation 2 (CCM2) scaffold protein significantly impacted the adhesion, polarity, spreading, and migratory abilities of epicardial cells, thereby impeding cardiac development and injury repair [87].

Epicardial cells also regulate the local microenvironment by secreting a variety of cytokines and chemokines to promote cardiomyocyte survival and functional recovery. Previous studies revealed that human amniotic epithelial stem cells (hAESCs) were able to secrete antioxidant, anti-fibrotic, and pro-angiogenic factors such as matrix metalloproteinase-9 (MMP-9), VEGF, and angiotensin, which regulate the inflammatory response and enhance tissue repair following MI [88,89]. Furthermore, epicardial cells are indispensable for neovascularization post-MI by promoting the proliferation and migration of vascular ECs through the secretion of factors like VEGF, accelerating the revascularization of the infarcted area [86]. Through single-cell RNA sequencing, Li et al. found that hAESCs could significantly reduce neutrophil and inflammatory macrophage infiltration post-MI and induce the polarization of CD4^+^CD8^+^ double-positive T-cells [88].

These studies, taken collectively, are a key determinant of epicardial cells in post-MI repair through mechanisms such as differentiation, cytokine secretion, immune response modulation, and neovascularization promotion, providing a theoretical foundation for the development of novel strategies for cardiac repair.

#### 3.2.4. Immune Cells

In recent years, it has been found that macrophages play a key role in damage repair after MI, and their functions and mechanisms vary according to the age of the individual, especially in neonatal, adult, and geriatric stages, showing significant differences.

In the neonatal stage, macrophages directly promote cardiomyocytes’ proliferation through the secretion of cytokines such as oncostatin M (OSM) and the activation of the Jagged-1-Notch1 signaling pathway, resulting in the scarless regeneration of the heart [90,91,92]. These macrophages are primarily derived from the yolk sac and fetal liver and exhibit multiple functions, including supporting coronary artery development and cardiac electrical conduction [91].

In adulthood, macrophage function shifts towards mediating inflammatory responses and scar formation. Following MI in adult mice, macrophages primarily form scar tissue by secreting inflammatory factors and chemokines, which activate fibroblasts and promote collagen deposition [90]. However, genetic or pharmacological interventions that activate specific signaling pathways in macrophages such as the gp130-Src-Yap pathway can partially restore their pro-regenerative capacity [92]. In addition, transplantation of neonatal-derived macrophages to the infarcted myocardium of adult mice significantly enhances cardiac repair and cardiomyocyte proliferation [93].

In old age, the pro-regenerative capacity of macrophages is further diminished, characterized by a more pronounced inflammatory response and a tendency toward fibrosis. Aged macrophages are more likely to exhibit a pro-inflammatory phenotype following MI, which may be related to aging and metabolic changes in cardiomyocytes. Studies suggest that modulating the phenotype and function of aged macrophages could be a crucial strategy to improve MI repair in the elderly [91]. In this context, activating regenerative signaling pathways present in neonatal macrophages or transplanting regenerative-competent neonatal macrophages into the hearts of adult and elderly patients may offer new therapeutic directions for MI treatment [92,93].

#### 3.2.5. Endothelial Cells

ECs are of paramount importance in the multifaceted process of injury repair following MI, including vascular regeneration, inflammation regulation, ECM remodeling, and modulation of the immune microenvironment.

ECs are the most abundant cell type in the heart, accounting for more than 60% of cardiac cells in mice [94]. In response to myocardial injury, ECs undergo revascularization through migration and proliferation to provide essential nutrient and oxygen support for cardiomyocyte regeneration [95]. ECs secrete cytokines (e.g., SDF-1α) and chemokines, and both attract and interact with immune cells through expressing immunomodulatory ligands. They promote cardiac repair by engaging in bidirectional signaling, which regulates immune cell recruitment, localization and activation, ultimately promoting inflammation resolution and tissue repair, which is mediated by transcription factors controlling immunomodulatory gene expression [96]. Additionally, ECs contribute to early scar formation via endothelial–mesenchymal transition, synthesizing and depositing ECM components. While crucial for initial wound healing, this process may trigger myocardial stiffness and dysfunction if dysregulated [97]. In terms of immunomodulation, ECs regulate the polarization state of inflammatory cells, particularly macrophages, promoting their transformation to a reparative phenotype [98].

Notably, ECs exhibit a staged response following MI: in the early phase, metabolic and inflammatory reactions prevail, with transcriptomic analysis revealing significant metabolic shifts and cell cycle initiation in ECs. During the middle phase, angiogenesis and fibrosis become prominent, while genes associated with ECM synthesis and angiogenesis in ECs are upregulated. Inflammatory and fibrotic characteristics are maintained in the long term. This temporal response suggests that precise interventions targeting ECs could optimize cardiac repair and improve post-MI prognosis [99]. These dynamic changes not only impact short-term repair but may also contribute to long-term vascular dysfunction and heart failure.

Furthermore, multiple programmed cell death (PCD) types, including apoptosis, necrosis, pyroptosis, ferroptosis, and autophagy, are activated in ECs following MI, contributing significantly to the pathological process. Developing therapeutic strategies targeting these PCD pathways, such as inhibition of apoptosis or modulation of autophagy, may offer new directions for the treatment of MI [100].

#### 3.2.6. Smooth Muscle Cells

Cardiac repair following MI is a complex process that involves the coordinated action of multiple cell types, during which SMCs emerge as pivotal contributors. SMCs contribute to cardiac repair via diverse mechanisms, including ECM synthesis and remodeling, anti-apoptotic actions, mechanical support, and regulation of inflammation.

First, SMCs maintain cardiac structural integrity by secreting ECM components (e.g., collagen and fibronectin) and influence cardiac remodeling by regulating the balance between matrix metalloproteinases (MMPs) and tissue-inhibitory factors (TIMPs) [101,102,103]. In addition, SMCs exhibit significant anti-apoptotic properties under ischemic conditions. They protect cardiomyocytes by secreting anti-apoptotic factors such as basic fibroblast growth factor (bFGF) and HGF [103]. This paracrine effect enhances the survival of SMCs and boosts the viability of neighboring cells, thereby alleviating ischemic injury [104]. SMCs also provide significant mechanical support during post-MI repair. It has been shown that SMC sheets are able to tightly attach to the infarcted area, reducing the expansion and thinning through their mechanical strength. This supportive function facilitates the maintenance of cardiac geometry and reduces cardiac load, thereby ultimately improving cardiac function [101]. Moreover, SMCs regulate the inflammatory response and recruitment of immune cells by secreting a variety of growth factors and cytokines. This regulatory effect facilitates the removal of necrotic tissue and promotes tissue repair [94]. The multifaceted roles of SMCs in post-MI repair highlight their potential as therapeutic targets for cardiac repair. Modulating their ECM synthesis, anti-apoptotic properties, mechanical support and inflammatory regulatory functions could lead to the development of novel therapeutic strategies. These strategies may optimize the cardiac repair process and improve prognosis following MI.

## 4. Addressing Immunological and Safety Challenges in Cell Therapy for Myocardial Infarction

Cell therapy, as an emerging therapeutic strategy, shows great potential in the treatment of myocardial infarction. However, immune rejection, oncogenicity and long-term safety issues remain major obstacles to its clinical application. Immune rejection may result in transplanted cells being recognized and attacked by the host immune system, thereby reducing therapeutic efficacy or even triggering an inflammatory response. For example, studies have shown that allogeneic cell transplantation may trigger a T-cell-mediated immune response, leading to the reduced survival of transplanted cells [105]. In addition, there is a potential risk of tumorigenicity associated with cell therapy, especially when using iPSCs or ESCs, which may form teratomas in vivo [106]. Long-term safety issues are also a major concern for cell therapy, including long-term cell survival, function, and potential effects on host tissues.

To address these issues, researchers are exploring a variety of strategies. For example, cells have been modified through gene editing techniques to enable them to evade recognition by the immune system, thereby reducing the risk of immune rejection [107]. In addition, strategies using autologous cell sources, such as cells derived from iPSCs, can be effective in avoiding the problem of immune rejection [105]. In terms of tumorigenicity, optimizing the cell preparation and differentiation process to ensure the purity and differentiation of cells can significantly reduce the risk of tumorigenicity [106]. Meanwhile, long-term follow-up studies and clinical trials are ongoing to assess the long-term safety and efficacy of cell therapy [108].

In conclusion, despite the immunological and safety challenges of cell therapy in the treatment of myocardial infarction, these issues are expected to be resolved through continued research and technological innovation, thus advancing the clinical use of cell therapy.

To clarify more explicitly, we comprehensively summarize repair mechanisms of the aforementioned cells (Table 1).

## 5. Challenges and Prospects of Research on Cell Therapy Mechanisms

Cell therapy, as a cutting-edge field in medical research, encounters numerous challenges in elucidating its underlying mechanisms. Paramount among these is the intricate mechanism between cells. Cells do not exist in isolation, but they interact with each other through multiple signaling pathways, cytokines, and cell-to-cell contacts to form a highly complex dynamic network. Such complex interactions make it challenging to fully understand the specific mechanisms of cell therapy, particularly the synergistic effects in multicellular environments of different origin and nature. In addition, this line of study is further complicated by several factors: cellular heterogeneity, variations in microenvironments, and differences in genetic backgrounds among individuals. These factors not only affect the efficacy of cell therapy but may also lead to unpredictable side effects.

Despite the numerous challenges, the study of cellular therapeutic mechanisms holds vast potential for the future. A deep exploration of the intricate mechanism between cells will lay a solid theoretical foundation for future clinical therapeutic research. By elucidating the interactions and regulatory mechanisms between cells, better design and optimization of cell therapy strategies can be provided for clinical use, thereby enhancing the precision and effectiveness of treatments and improving patient prognosis. Meanwhile, technological advances, such as single-cell sequencing, gene editing and bioinformatics analysis, are expected to offer a more comprehensive understanding of the molecular mechanisms underlying cell therapy, paving the way for personalized medicine. Future research will not only promote the application of cell therapy in cardiovascular disease, cancer, and neurodegenerative diseases, but also bring new hope to regenerative medicine and tissue engineering, opening up a new avenue in human health.

## Figures and Tables

**Table 1:** Cellular repair mechanisms following MI.

Cell Type	Summary of Functions	References
Mesenchymal Stem Cells	1. Proliferate and differentiate into cardiomyocytes and ECs to assist angiogenesis and protect cardiomyocytes	[29,33,34]
	2. Secrete exosomes containing miR-214 to promote neovascularization	[37]
	3. Overexpress GATA4 and MYOCD to reduce cardiomyocyte apoptosis	[38]
Bone Marrow Mononuclear Cells	1. Differentiate into immune cells and ECs	[45]
	2. Secrete angiogenic factors in a paracrine manner	[46,47,48]
	3. Immunosuppression: reduce T cell proliferation and inflammatory response	[49,50,51]
Adipose-Derived Stem Cells	1. Directly differentiate into cardiomyocytes, vascular SMCs and ECs to restore cardiac function after MI	[53,54]
	2. Secrete a variety of cytokines (such as VEGF, HGF, IGF-1) to promote angiogenesis, reduce apoptosis and inhibit fibrosis	
Exogenous Cardiac Stem Cells	1. Secrete anti-inflammatory factors and promote angiogenesis	[56]
	2. Regulate the biological behavior of cardiomyocytes and fibroblasts, inhibit the process of fibrosis, and promote myocardial repair	[57]
	3. Directly differentiate into other cells and participate in the process of inflammation repair	[57]
Fibroblasts	1. Transform into myofibroblasts to form fibrotic scars	[75]
	2. Regulate electrophysiological properties and intercellular signaling	[73]
	3. Secrete cytokines	[74]
Cardiomyocytes	1. Proliferate to promote myocardial regeneration	[79]
	2. Secrete cytokines in a paracrine manner	[80]
	3. Regulate the cell cycle	[79]
Epicardial Cells	1. Differentiate into cardiovascular cells	[86]
	2. Secrete cytokines and chemokines	[88]
	3. Regulate inflammatory responses and promote angiogenesis	[86]
Immune Cells	1. Secrete OSM and activate the Jagged-1-Notch1 signaling pathway to promote cardiomyocyte proliferation and scar-free regeneration 2. Participate in the inflammatory response and scarring 3. Modulate its phenotype and function to improve repair	[90,91]
Endothelial Cells	1. Migrate and proliferate to promote vascular regeneration	[95]
	2. Secrete cytokines and chemokines to regulate inflammation	[96]
	3. Participate in ECM remodeling and immune microenvironment regulation	[97]
Smooth Muscle Cells	1. Participate in the synthesis and remodeling of the extracellular matrix	[101,102,103]
	2. Exhibit anti-apoptotic properties	[103]
	3. Provide mechanical support	[101]
	4. Regulate inflammatory responses and immune cell recruitment	[104]

## Abbreviations

The following abbreviations are used in this manuscript:

ADSCs	Adipose-derived stem cells
BADSCs	Brown adipose tissue stem cells
bFGF	Basic fibroblast growth factor
ECs	Endothelial cells
EDCs	Explant-derived cardiac cells
EMT	Epithelial–mesenchymal transition
EPCs	Endothelial progenitor cells
Fbs	Fibroblasts
FGF	Fibroblast growth factor
hAESCs	Human amniotic epithelial stem cells
HGF	Hepatocyte growth factor
HSCs	Hematopoietic stem cells
IGF-1	Insulin-like growth factor-1
iPS-CMs	Induced pluripotent stem cells-derived cardiomyocytes
IPSCs	Induced pluripotent stem cells
KLF4	Krüpple-like factor 4
LepR+	Leptin receptor+
LPAR4	Lysophosphatidic acid receptor 4
LVEF	Left ventricular ejection fraction
LVFS	Left ventricular fractional shortening
MI	Myocardial infarction
MIF	Macrophage migration inhibitory factor
miR-10α	MicroRNA-10α
MMP-9	Matrix metalloproteinase-9
MMPs	Matrix metalloproteinases
MSCs	Mesenchymal stem cells
MYOCD	Myocardin
NADH	Nicotinamide adenine dinucleotide hydrate
OSM	Oncostatin M
p16INK4a	Cyclin-dependent kinase inhibitor 2A
PCD	Programmed cell death
PSCs	Pluripotent stem cells
ROS	Reactive oxygen species
SA-β-gal	Senescence-associated β-galactosidase
SCF	Stem cell factor
SDF-1	Stromal cell-derived factor 1
SMCs	Smooth muscle cells
SOX17	SRY-box transcription factor 17
STEMI	ST-segment elevation myocardial infarction
TIMPs	Tissue-inhibitory factors
VEGF	Vascular endothelial growth factor
ΔΨm	Mitochondrial membrane potential
